# Supplementary material for: “Hands-On” and “Hands-Off” Physiotherapy Treatments in Fibromyalgia Patients: A Systematic Review and Meta-Analysis
Source: Biomedicines. 2024 Oct 21;12(10):2412. doi: 10.3390/biomedicines12102412 (PMC11506077; doi:10.3390/biomedicines12102412)
Supplement: Supplementary file 1 [file biomedicines-12-02412-s001.zip › File S2-Search strategy.pdf]

Supplementary Material 2. Search strategy.

DATABASE	SEARCH STRATEGY
PubMed	"Fibromyalgia"[Mesh]<br>"Fibromyalgi*" OR "Fibromyositis" OR "Fibrositis" OR "FMS"<br>#1 OR #2<br>"Physical Therapy Modalities"[Mesh] OR "Exercise Therapy"[Mesh] OR "Rehabilitation"[Mesh]<br>"manual therap*" OR "exercise*" OR "physiotherap*" OR "training" OR "physical therap*"<br>#4 OR #5<br>"randomized controlled trial" [pt] OR "controlled clinical trial" [pt] OR "randomized" [tiab] OR "placebo" [tiab] OR "clinical trials as topic" [mesh] OR "randomly" [tiab] OR "trial" [ti]<br>animals [mh] NOT humans [mh]<br>#7 NOT #8<br>#3 AND #6 AND #9
Central	MeSH descriptor: [Fibromyalgia] explode all trees<br>("Fibromyalgi*" OR "Fibromyositis" OR "Fibrositis" OR "FMS"):ti,ab,kw<br>MeSH descriptor: [Physical Therapy Modalities] explode all trees<br>MeSH descriptor: [Exercise Therapy] explode all trees<br>MeSH descriptor: [Rehabilitation] explode all trees<br>((manual NEXT therap*) OR exercise* OR physiotherap* OR training OR (physical NEXT therap*)):ti,ab,kw<br>#1 OR #2<br>#3 OR #4 OR #5 OR #6<br>#7 AND #8
Embase	'fibromyalgia'/exp OR 'fibromyalgia':ti,ab,kw OR 'polymyositis'/exp OR polymyositis:ti,ab,kw OR 'fibrositis':ti,ab,kw OR 'fms':ti,ab,kw<br>'manual therapy'/exp OR 'manual therap*':ti,ab,kw OR 'exercise therapy'/exp OR 'exercis*':ti,ab,kw OR 'physiotherapy'/exp OR 'physiotherap*':ti,ab,kw OR 'training'/exp OR training:ti,ab,kw OR 'physical therap*':ti,ab,kw<br>('randomized controlled trial'/de OR 'controlled clinical trial'/de OR random*:ti,ab OR 'randomization'/de OR 'intermethod comparison'/de OR placebo:ti,ab OR compare:ti OR compared:ti OR comparison:ti OR ((evaluated:ab OR evaluate:ab OR evaluating:ab OR assessed:ab OR assess:ab) AND (compare:ab OR compared:ab OR comparing:ab OR comparison:ab)) OR ((open NEXT/1 label):ti,ab) OR (((double OR single OR doubly OR singly) NEXT/1 (blind OR blinded OR blindly)):ti,ab) OR 'double blind procedure'/de OR ((parallel NEXT/1 group*):ti,ab) OR crossover:ti,ab OR 'cross over':ti,ab OR (((assign* OR match OR matched OR allocation) NEAR/6 (alternate OR group OR groups OR intervention OR interventions OR patient OR patients OR subject OR subjects OR participant OR participants)):ti,ab) OR assigned:ti,ab OR allocated:ti,ab OR ((controlled NEAR/8 (study OR design OR trial)):ti,ab) OR volunteer:ti,ab OR volunteers:ti,ab OR 'human experiment'/de OR trial:ti) NOT (((random* NEXT/1 sampl* NEAR/8 ('cross section*' OR questionnaire* OR survey OR surveys OR database OR databases)):ti,ab) NOT ('comparative study'/de OR 'controlled study'/de OR 'randomised controlled':ti,ab OR 'randomized controlled':ti,ab OR 'randomly assigned':ti,ab) OR ('cross-sectional study' NOT ('randomized controlled trial'/de OR 'controlled clinical study'/de OR 'controlled study'/de OR 'randomised controlled':ti,ab OR 'randomized controlled':ti,ab OR 'control group':ti,ab OR 'control groups':ti,ab)) OR ('case control*':ti,ab AND random*:ti,ab NOT ('randomised controlled':ti,ab OR 'randomized controlled':ti,ab)) OR ('systematic review':ti NOT (trial:ti OR study:ti)) OR (nonrandom*:ti,ab NOT random*:ti,ab) OR 'random field*':ti,ab OR (('random cluster' NEAR/4 sampl*):ti,ab) OR (review:ab AND review:it NOT trial:ti) OR ('we searched':ab AND (review:ti OR review:it)) OR

	'update review':ab OR ((databases NEAR/5 searched):ab) OR ((rat:ti OR rats:ti OR mouse:ti OR mice:ti OR swine:ti OR porcine:ti OR murine:ti OR sheep:ti OR lambs:ti OR pigs:ti OR piglets:ti OR rabbit:ti OR rabbits:ti OR cat:ti OR cats:ti OR dog:ti OR dogs:ti OR cattle:ti OR bovine:ti OR monkey:ti OR monkeys:ti OR trout:ti OR marmoset*:ti) AND 'animal experiment'/de) OR ('animal experiment'/de NOT ('human experiment'/de OR 'human'/de)))<br>#1 AND #2 AND #3
