# Supplementary material for: “Hands-On” and “Hands-Off” Physiotherapy Treatments in Fibromyalgia Patients: A Systematic Review and Meta-Analysis
Source: Biomedicines. 2024 Oct 21;12(10):2412. doi: 10.3390/biomedicines12102412 (PMC11506077; doi:10.3390/biomedicines12102412)
Supplement: Supplementary file 1 [file biomedicines-12-02412-s001.zip › File S3-Excluded studies.pdf]

Publication Year	Author	Title	Journal Title	Exclusion reason
2023	Schulze, N.B.B.; Barreto, T.D.N.P.; Alencar, G.G.; da Silva, T.A.; Duarte, A.L.B.P.; Ranzolin, A.; Siqueira, G.R.	The effect of myofascial release of the physiological chains on the pain and health status in patients with fibromyalgia, compared to passive muscle stretching and a control group: a randomized controlled clinical trial	Disability and rehabilitation	wrong intervention
2023	Rodríguez-Mansilla, J.; Mejías-Gil, A.; Garrido-Ardila, E.M.; Jiménez-Palomares, M.; Montanero-Fernández, J.; González-López-Arza, M.V.	Effects of an Exercise for Well-Being and Physical Training Programme on Muscle Strength, Range of Movement, Respiratory Capacity and Quality of Life in Women with Fibromyalgia: A Randomized Controlled Trial	Journal of Clinical Medicine	wrong intervention
2022	Ducamp, P.; Sichère, P.; Gayum, H.; Dubourg, K.; Roques, C.-F.	Republication of: “Therapeutic Patient Education for Fibromyalgia during Spa Therapy: The FiETT Randomized Controlled Trial”	Douleurs	wrong study design
2022	Capodaglio, P.; Fontana, J.; Varallo, G.	Whole-Body Cryostimulation in Fibromyalgia	Archives of Physical Medicine and Rehabilitation	conference abstract
2022	İlgen, U.	On "Effectiveness of High-Frequency Transcranial Magnetic Stimulation and Physical Exercise in Women With Fibromyalgia: A Randomized Controlled Trial."	Physical therapy	wrong study design
2022	Tomas-Carus, P.; Biehl-Printes, C.; del Pozo-Cruz, J.; Parraca, J.A.; Folgado, H.; Pérez-Sousa, M.A.	Effects of respiratory muscle training on respiratory efficiency and health-related quality of life in sedentary women with fibromyalgia: a randomised controlled trial	Clinical and Experimental Rheumatology	wrong intervention
2022	Dailey, D.L.; Vance, C.G.; Chimenti, R.L.; Franck, C.; Post, A.; Johnson, E.; Costigan, M.; Koepp, M.; Huff, T.; Van Gorp, B.; Ecklund, D.; Bayman, E.O.; Archer, K.R.; Zimmerman, B.; Crofford, L.J.; Sluka, K.A.	Physical Therapist Training Challenges for an Embedded Pragmatic Trial: Fibromyalgia TENS in Physical Therapy Study	Journal of Pain	conference abstract

2022	Ducamp, P.; Sichère, P.; Gayum, H.; Dubourg, K.; Roques, C.-F.; Journot, V.	Therapeutic Patient Education for Fibromyalgia during Spa Therapy: The FiETT Randomized Controlled Trial	International Journal of Environmental Research and Public Health	conference abstract
2017	Pastor-Mira, M.Á.; López-Roig, S.; Peñacoba, C.; Lledó-Boyer, A.	Feasibility of using implementation intentions to increase physical exercise walking in women with fibromyalgia	Revista de la Sociedad Espanola del Dolor	other languages
2013	Morgan, N; Morgan, L; Price, L L; Wang, C	Mindfulness is associated with psychological symptoms, self-efficacy and quality of life among patients with fibromyalgia	Arthritis and Rheumatism	conference abstract
2011	Schmidt, Stefan; Grossman, Paul; Schwarzer, Barbara; Jena, Susanne; Naumann, Johannes; Walach, Harald	Treating fibromyalgia with mindfulness-based stress reduction: Results from a 3-armed randomized controlled trial	Pain	uncomplete d study
2018	Trivedi, V; Mishra, P	The efficacy of classical indian yoga in the treatment of fibromyalgia: A randomized controlled trial	Internal Medicine Journal	uncomplete d study
2014	Özer, Z; Şahin, Ö; Özer, P K; Tunc ̧ay, M S	Assessment of effectiveness of balneotherapy and physical agents in fibromyalgia syndrome	Clinical and Experimental Rheumatology	wrong study design
2017	Jiao, J; Russell, I J; Wang, W; Zhao, Y.-Y.; Zhang, R.-M.; Wang, J; Jiang, Q	Is lu eight-brocades exercise beneficial for patients with fibromyalgia?	Arthritis and Rheumatology	conference abstract
2016	Wang, C; Schmid, C; Fielding, R A; Harvey, W F; Price, L L; Driban, J B; Reid, K; Kalish, R A; Rones, R; McAlindon, T E	Tai CHI is more effective than aerobic exercise in treating fibromyalgia: A randomized controlled trial	Arthritis and Rheumatology	conference abstract
2018	Ozyasar, A; Karatepe, A G	Effectiveness of 8-weeks supervised and nonsupervised aerobic exercise programson clinic findings, functional status and qality of life in the patients with fibromyalgia syndrome	Annals of the Rheumatic Diseases	conference abstract

2019	Yavuz, Hadi; Şahinkaya, Türker; Ketenci, Ayşegül; Metin, Gökhan	Ab1379-Hpr Effects of Different Exercise Models on Pain, Functionality, Balance, Proprioception and Cognition Features of Patients Diagnosed With Fibromyalgia	Annals of the Rheumatic Diseases	conference abstract
2010	Carbonell-Baeza, Ana; Aparicio, Virginia A; Martins-Pereira, Clelia M; Gatto-Cardia, Claudia M; Ortega, Francisco B; Huertas, Francisco J; Tercedor, Pablo; Ruiz, Jonatan R; Delgado-Fernandez, Manuel	Efficacy of Biodanza for treating women with fibromyalgia.	Journal of alternative and complementary medicine (New York, N.Y.)	wrong study design
2019	Villafaina, Santos; Collado-Mateo, Daniel; Fuentes, Juan Pedro; Rohlfs-Domínguez, Paloma; Gusi, Narcís	Effects of Exergames on Brain Dynamics in Women with Fibromyalgia: A Randomized Controlled Trial	Journal of Clinical Medicine	wrong outcome
2020	Keskin, A.; Basakci Calik, B.; Gur Kabul, E.; Cobankara, V.	Ab0954 Is Connective Tissue Massage Effective in Individuals With Fibromyalgia?	Annals of the Rheumatic Diseases	conference abstract
2019	Alventosa, Ruth Izquierdo; Inglés, Marta; Amador, Sara Cortés; Gimeno-Mallench, Lucía; Vidal, Àngels; Chirivella, Javier; Kropotov, Juri D.; Serra-Añó, Pilar	Ab1367-Hpr Effectiveness of the Hyperbaric Treatment on the Perceived Pain, Fatigue and Functionality of Women With Fibromyalgia	Annals of the Rheumatic Diseases	conference abstract
2013	Bettoni, Lorenzo; Bonomi, Felice Giulio; Zani, Viviana; Manisco, Luigia; Indelicato, Annamaria; Lanteri, Patrizia; Banfi, Giuseppe; Lombardi, Giovanni	Effects of 15 consecutive cryotherapy sessions on the clinical output of fibromyalgic patients	Clinical Rheumatology	wrong study design
2018	Haugmark, Trond; Hagen, Kåre Birger; Provan, Sella Aarrestad; Bærheim, Elisebeth; Zangi, Heidi A.	Effects of a community-based multicomponent rehabilitation programme for patients with fibromyalgia: Protocol for a randomised controlled trial	BMJ Open	uncomplete d study
2010	Ang, D C; Kaleth, A S; Bigatti, S M; Mazzuca, S A; Saha, C K; Bandy, R W	A randomized attention-controlled study of motivational interviewing to encourage exercise in fibromyalgia: Week 12 interim analysis	Arthritis and Rheumatism	conference abstract

2014	Nugraha, Boya; Korallus, Christoph; Dörffer, Denise; Dipl Psych, Sarah Zastrutzki; Jasper, Stefanie; Jäger, Bürkard; GutenBrunner c, Christoph	No. 373 Aerobic Exercise Cognitive Behavioural Therapy and Combination of Treatment in Fibromyalgia Syndrome Patients: A Randomized Control Trial (Effect on Mood Related Disorder-A Preliminary Result)	Pm&R	conference abstract
2020	Rulleau, T.; Planche, L.; Etcheverrigaray, F.; Dorion, A.; Kacki, N.; Miot, M.; Liaigre, A.; Ganem, Y.; Schmidt, A.; Taddéi, F.; Acapo, S.; Nizard, J.; Pluchon, Y. M.	Comparison of patient-led, fibromyalgia-orientated physical activity and a non-specific, standardised 6-month physical activity program on quality of life in individuals with fibromyalgia: A protocol for a randomised controlled trial	Trials	uncomplete d study
2005	Yousefi, Pouran; Coffey, John	Clinical inquiries. For fibromyalgia, which treatments are the most effective?	The Journal of family practice	wrong study design
2005	Kashikar-Zuck, Susmita; Swain, Nicole F.; Jones, Benjamin A.; Graham, T. Brent	Efficacy of cognitive-behavioral intervention for juvenile primary fibromyalgia syndrome	Journal of Rheumatology	wrong study design
2019	Carrera, B G; Segura-Jiménez, V; Acosta-Manzano, P; Estevez-Lopez, F; Cosic, M B; Aparicio, V A; Carbonell-Baeza, A; Delgado-Fernández, M	Comparative effectiveness of land and water-based exercise on quality of life of patients with fibromyalgia: Preliminary findings from the Al-Ándalus randomised controlled trial	Annals of the Rheumatic Diseases	conference abstract
2009	Williams, D A; Ambrose, K; Skalski, L; Muroff, J; Zwinck, L; Clauw, D	Improving internal locus of pain control in fibromyalgia	Arthritis and Rheumatism	conference abstract
2020	Norouzi, Ebrahim; Hosseini, Fatemeh Sadat; Vaezmosavi, Mohammad; Gerber, Markus; Pühse, Uwe; Brand, Serge	Zumba dancing and aerobic exercise can improve working memory, motor function, and depressive symptoms in female patients with Fibromyalgia	European Journal of Sport Science	wrong outcome
2018	Yuan, S L K; Marques, A P	Effectiveness of profibro mobile app on quality of life, symptoms and self-care agency in patients with fibromyalgia: A randomised, single-blind trial	Annals of the Rheumatic Diseases	conference abstract
2012	Wong, J B; Wang, C	Cost-effectiveness of Tai CHI mind-body exercise for the treatment of fibromyalgia	Arthritis and Rheumatism	conference abstract

2020	Swar, W K	Effect of underwater exercises on treating postmenopausal fibromyalgia symptoms	European Journal of Molecular and Clinical Medicine	wrong study design
2012	Wright, C; Carson, J; Carson, K; Bennett, R; Mist, S; Jones, K	P02.193. Yoga of awareness: a randomized trial in fibromyalgia: post intervention and 3 month follow up results	BMC Complementary and Alternative Medicine	conference abstract
2011	Carbonell-Baeza, Ana; Aparicio, Virginia A.; Ortega, Francisco B.; Cuevas, Ana M.; Alvarez, Inmaculada C.; Ruiz, Jonatan R.; Delgado-Fernandez, Manuel	Does a 3-month multidisciplinary intervention improve pain, body composition and physical fi tness in women with fibromyalgia?	British Journal of Sports Medicine	wrong study design
2018	Chung, M; Beauchesne, A; Fu, Z; Price, L L; Wang, C	Association of diet quality with overall fibromyalgia impact, and psychosocial and quality of life outcomes in women with fibromyalgia	Arthritis and Rheumatology	conference abstract
2019	Cagla, C B; Keskin, A; Kabul, E G; Calik, B B; Aslan, U B; Karasu, U	The effects of clinical pilates training in patients with fibromyalgia: A randomized controlled trial	Annals of the Rheumatic Diseases	conference abstract
2013	Latorre, Pedro Angel; Santos, Maria Aparecida; Heredia-Jiménez, Jose Maria; Delgado-Fernández, Manuel; Soto, Victor Manuel; Mañas, Alfonso; Carbonell-Baeza, Ana	Effect of a 24-week physical training programme (in water and on land) on pain, functional capacity, body composition and quality of life in women with fibromyalgia	Clinical and Experimental Rheumatology	wrong study design
2009	Geler Külcü, Duygu; Gülşen, Gülçin; Geler Kulcu, D; Gulsen, G	Effect of physical therapy program on insomnia severity in a patient population with fibromyalgia syndrome	Turkiye Fiziksel Tip ve Rehabilitasyon Dergisi	other languages

2011	Carbonell-Baeza, A.; Aparicio, V. A.; Chillón, P.; Femia, P.; Delgado-Fernández, M.; Ruiz, J. R.	Effectiveness of multidisciplinary therapy on symptomatology and quality of life in women with fibromyalgia	Clinical and Experimental Rheumatology	wrong study design
2017	Collado-Mateo, Daniel; Dominguez-Muñoz, Francisco J.; Adsuar, Jose C.; Merellano-Navarro, Eugenio; Gusi, Narcis	Exergames for women with fibromyalgia: A randomised controlled trial to evaluate the effects on mobility skills, balance and fear of falling	PeerJ	wrong outcome
2021	Pearson, Jennifer; Coggins, Jessica; Lenguerrand, Erik; Derham, Sandi; Russell, Julie; Walsh, Nicola E.; Cramp, Fiona	A feasibility randomised controlled trial of a fibromyalgia self-management programme in a community setting with a nested qualitative study (FALCON): Study protocol	Musculoskeletal Care	uncompleted study
2014	Wolcott, E; Harvey, W F; Price, L L; Driban, J B; Morgan, N; Morgan, L; Wang, C	Mindfulness is associated with symptom severity and pain impact in patients with fibromyalgia	Arthritis and Rheumatology	conference abstract
2011	Van Ittersum, M	Does pain physiology education change illness perceptions in patients with fibromyalgia? A randomised controlled trial with six months follow up	Physiotherapy (United Kingdom)	conference abstract
2020	Rivas Neira, S; Pasqual Marques, A; Vivas Costa, J	Is aquatic therapy more effective than landbased therapy in reducing pain of women with fibromyalgia?	Annals of the Rheumatic Diseases	wrong intervention
2018	Santos, Vinicius Souza Dos Souza Dos; Zortea, Maxciel; Alves, Rael Lopes; Naziazeno, Cátia Cilene Dos Santos; Saldanha, Júlia Schirmer; Carvalho, Sandra da Conceição Ribeiro de; Leite, António Jorge da Costa; Torres, Iraci Lucena da Silva; Souza, Andressa de; Calvetti, Prisla Ücker; Fregni, Felipe; Caumo, Wolnei	Cognitive effects of transcranial direct current stimulation combined with working memory training in fibromyalgia: a randomized clinical trial.	Scientific reports	wrong outcome

2011	Mendonca, Mariana E; Santana, Marcus B; Baptista, Abrahão F; Datta, Abhishek; Bikson, Marom; Fregni, Felipe; Araujo, Cintia P	Transcranial DC stimulation in fibromyalgia: optimized cortical target supported by high-resolution computational models.	The journal of pain	wrong outcome
2018	Vance, Carol Gt; Chimenti, Ruth L; Dailey, Dana L; Hadlandsmyth, Katherine; Zimmerman, M Bridget; Geasland, Katharine M; Williams, Jonathan M; Merriwether, Ericka N; Alemo Munters, Li; Rakel, Barbara A; Crofford, Leslie J; Sluka, Kathleen A	Development of a method to maximize the transcutaneous electrical nerve stimulation intensity in women with fibromyalgia.	Journal of pain research	wrong study design
2020	Gavilán Carrera, B.; Segura-Jiménez, V.; Acosta-Manzano, P.; Borges Cosic, M.; Estévez-López, F.; Delgado-Fernández, M.	Fri0647 Comparative Effectiveness of Land and Water-Based Exercise Programs on Fatigue in Women With Fibromyalgia: Preliminary Findings From the Al-Ándalus Randomised Controlled Trial.	Annals of the Rheumatic Diseases	conference abstract
2019	Grieve, R.; Russell, J.; Derham, S.; Palmer, S.	Self-applied soft tissue therapy for fibromyalgia syndrome: a randomised controlled feasibility study	Physiotherapy	conference abstract
2019	Estevez-Lopez, Fernando; Alvarez-Gallardo, Inmaculada C.; Segura-Jiménez, Víctor; Cosic, Milkana Borges; Acosta-Manzano, Pedro; Carrera, Blanca Gavilán; Carbonell-Baeza, Ana; Delgado-Fernández, Manuel; Aparicio, Virginia A.	Fri0709-Hpr Effects of Land- and Water-Based Exercise Interventions on Pain in People With Fibromyalgia: a Preliminary Report From the Al-Ándalus Randomised Controlled Trial	Annals of the Rheumatic Diseases	conference abstract
2014	Anderson, Joel G.; Kebaish, Samy A.; Lewis, Janet E.; Taylor, Ann Gill	Effects of cranial electrical stimulation on activity in regions of the basal ganglia in individuals with fibromyalgia	Journal of Alternative and Complementary Medicine	wrong study design
2012	Hamnes, Bente; Mowinckel, Petter; Kjeken, Ingvild; Hagen, Kåre B.	Effects of a one week multidisciplinary inpatient self-management programme for patients with fibromyalgia: A randomised controlled trial	BMC Musculoskeletal Disorders	wrong study design

2019	Castelo-Branco, Luis; Uygur Kucukseymen, Elif; Duarte, Dante; El-Hagrassy, Mirret M.; Bonin Pinto, Camila; Gunduz, Muhammed Enes; Cardenas-Rojas, Alejandra; Pacheco-Barrios, Kevin; Yang, Yiling; Gonzalez-Mego, Paola; Estudillo-Guerra, Anayali; Candido-Santos, Ludmilla; Mesia-Toledo, Ines; Rafferty, Haley; Caumo, Wolnei; Fregni, Felipe	Optimised transcranial direct current stimulation (tDCS) for fibromyalgia - Targeting the endogenous pain control system: A randomised, double-blind, factorial clinical trial protocol	BMJ Open	uncompleted study
2013	Kuru, O; Yildiz, N; Bilgici, A	The effectiveness of aerobic exercise and education in fibromyalgia syndrome	International Journal of Rheumatic Diseases	wrong study design
2014	Zhang, Y; Price, L L; Morgan, N; Morgan, L; Wang, C	Mindfulness is associated with sleep quality among patients with fibromyalgia	Arthritis and Rheumatology	wrong study design
2000	Meiworm, L.; Jakob, E.; Walker, U. A.; Peter, H. H.; Keul, J.	Patients with fibromyalgia benefit from aerobic endurance exercise	Clinical Rheumatology	wrong study design
2014	Kibar, S.; Yıldız, E.; Ay, S.; Evcik, D.	THU0324 Is Balance Training Exercise Program Effective in Fibromiyalgia Syndrome?	Annals of the Rheumatic Diseases	conference abstract
2011	Van Oosterwijck, J; Nijs, J; Meeus, M; De Schryver, M; Pascal, A; Paul, L	Can pain physiology education change pain cognitions and descending nociceptive processing in fibromyalgia? A randomised controlled trial	Physiotherapy (United Kingdom)	conference abstract
2013	Carbonario, F.; Matsutani, L. A.; Yuan, S. L.K.; Marques, A. P.	Effectiveness of high-frequency transcutaneous electrical nerve stimulation at tender points as adjuvant therapy for patients with fibromyalgia	European Journal of Physical and Rehabilitation Medicine	wrong study design
2012	Demirbag, C; Oguzoncul, F	Effects of education and exercise on pain, depression and quality of life in patients diagnosed with Fibromyalgia	HealthMED	wrong study design

2012	Carbonell-Baeza, Ana; Ruiz, Jonatan R.; Aparicio, Virginia A.; Ortega, Francisco B.; Munguía-Izquierdo, Diego; Álvarez-Gallardo, Inmaculada C.; Segura-Jiménez, Víctor; Camiletti-Moirán, Daniel; Romero, Alejandro; Estévez-Lápez, Fernando; Samos, Blanca; Casimiro, Antonio J.; Sierra, Ángela; Latorre, Pedro A.; Pulido-Martos, Manuel; Femia, Pedro; Pérez-Lápez, Isaac J.; Chillán, Palma; Girela-Reján, María J.; Tercedor, Pablo; Lucía, Alejandro; Delgado-Fernández, Manuel	Land- and water-based exercise intervention in women with fibromyalgia: The al-Andalus physical activity randomised controlled trial	BMC Musculoskeletal Disorders	wrong intervention
2010	Gowans, S E	Fibromyalgia: Increased regular physical activity as 'exercise' in fibromyalgia.	Nature reviews. Rheumatology	wrong study design
2021	Hernando-Garijo, Ignacio; Ceballos-Laita, Luis; Mingo-Gómez, María Teresa; Medrano-De-la-fuente, Ricardo; Estébanez-De-miguel, Elena; Martínez-Pérez, María Natividad; Jiménez-Del-barrio, Sandra	Immediate effects of a telerehabilitation program based on aerobic exercise in women with fibromyalgia	International Journal of Environmental Research and Public Health	wrong study design
1995	White, A	The fibromyalgia syndrome. Electroacupuncture is a potentially valuable treatment.	BMJ (Clinical research ed.)	uncomplete d study
2012	de Carvalho Pde, T; Leal-Junior, E C; Alves, A C; Rambo, C S; Sampaio, L M; Oliveira, C S; Albertini, R; de Oliveira, L V	Effect of low-level laser therapy on pain, quality of life and sleep in patients with fibromyalgia: study protocol for a double-blinded randomized controlled trial	Trials	uncomplete d study

2018	Vassalli, M.C.; Jones, A.; Natour, J.; Silva, R.; Vassalli, M.C.; Da Silva, R V T; Jones, A.; Natour, J.; Silva, R.	OP0277-HPR Evaluation of the effectiveness of a progressive resistance training program for patients with fibromyalgia: a randomised controlled trial	Advances in Rheumatology	conference abstract
2010	Jones, Kim D.	Nordic walking in fibromyalgia: A means of promoting fitness that is easy for busy clinicians to recommend	Arthritis Research and Therapy	wrong study design
2022	Mongini, T.; Gadaleta, G.	Body Posture Recognition in NMDs	Journal of Neuromuscular Diseases	wrong population
2023	Peterson, H.M.; Vela, K.L.; Barnado, A.; Patrick, A.E.	Developing electronic health record algorithms that accurately identify patients with juvenile idiopathic arthritis	Seminars in Arthritis and Rheumatism	wrong population
2023	Madani, Z.; Bagheri, S.; Elyasi, F.; Mobini, M.; Charati, J.Y.; Maleklou, F.; Ahmadi, H.	Efficacy of Stretching Exercises on Fibromyalgia Impact and Quality of Life in Patients under Medical Treatment: A Randomized Clinical Trial, 2021	Journal of Mazandaran University of Medical Sciences	wrong intervention
2023	Ramasawmy, P.; Khalid, S.; Petzke, F.; Antal, A.	P-21 Mindfully stimulating the brain: A randomized, sham-controlled and double-blinded pilot clinical trial combining mindfulness meditation and transcranial direct current stimulation in fibromyalgia	Clinical Neurophysiology	wrong intervention
2023	Meriwether, K.V.; Ravichandran, N.; Darley, C.J.; Panter, V.; Komesu, Y.M.	Centering Group Treatment for Women With Interstitial Cystitis/Bladder Pain Syndrome: A Prospective, Parallel-Group Cohort Study	Urogynecology	wrong population
2023	Karacay, B.C.; Sahbaz, T.; Ceylan, C.M.	The vicious cycle of physical inactivity, fatigue and kinesiophobia in patients with fibromyalgia syndrome	Reumatismo	wrong intervention
2023	Hung, C.-H.; Tsai, M.-H.; Wang, P.-S.; Liang, F.-W.; Hsu, C.-Y.; Lee, K.-W.; Fong, Y.-O.; Han, D.-S.; Lee, C.-H.; Lai, C.-L.; Chen, C.-C.	Oxidative stress involves phenotype modulation of morbid soreness symptoms in fibromyalgia	RMD Open	wrong intervention

2023	Ince, B.; Kara, M.; Erdem, I.; Yurdakul, O.V.; Erden, T.; Aydın, T.	Effectiveness of spinal manipulation in addition to pharmacological treatment in fibromyalgia: A blinded randomized trial	PM and R	wrong intervention
2023	Batrakoulis, A.; Jamurtas, A.Z.; Tsimeas, P.; Poulios, A.; Perivoliotis, K.; Syrou, N.; Papanikolaou, K.; Draganidis, D.; Deli, C.K.; Metsios, G.S.; Angelopoulos, T.J.; Feito, Y.; Fatouros, I.G.	Hybrid-type, multicomponent interval training upregulates musculoskeletal fitness of adults with overweight and obesity in a volume-dependent manner: A 1-year dose-response randomised controlled trial	European journal of sport science	wrong population
2023	Ferguson, O.N.; Mitchell, R.A.; Schaeffer, M.R.; Ramsook, A.H.; Dhillon, S.S.; Dominelli, P.B.; Molgat-Seon, Y.; Guenette, J.A.	Effects of Face Masks on the Multiple Dimensions and Neurophysiological Mechanisms of Exertional Dyspnea	Medicine and science in sports and exercise	wrong population
2023	Hessam, M.; Fathalipour, K.; Behdarvandan, A.; Goharpey, S.	The Effect of McGill Core Stability Training on Movement Patterns, Shooting Accuracy, and Throwing Performance in Male Basketball Players: A Randomized Controlled Trial	Journal of sport rehabilitation	wrong population
2023	Estrada-Marcén, N.C.; Casterad-Seral, J.; Montero-Marin, J.; Serrano-Ostáriz, E.	Can an Aerobic Exercise Programme Improve the Response of the Growth Hormone in Fibromyalgia Patients? A Randomised Controlled Trial	International Journal of Environmental Research and Public Health	wrong intervention
2023	Kemer, S.N.; Celik, H.İ.; Ozal, S.; Kafa, N.	The Effect of Kinesio Taping Over the Gluteal Muscles on Activity and Participation in Children with Unilateral Cerebral Palsy: A Preliminary Randomized Controlled Study	Iranian Journal of Pediatrics	wrong population
2023	Bayman, E.O.; Ecklund, D.; Sluka, K.; Berardi, G.; Dailey, D.	INCREASING THE GENERALIZABILITY OF PAIN STUDY RESULTS	Clinical Trials	wrong intervention
2023	Burgess, H.J.; Bahl, S.; Wilensky, K.; Spence, E.; Jouppi, R.J.; Rizvydeen, M.; Goldstein, C.; Kim, H.M.; Williams, D.A.; Burns, J.W.	A 4-Week Morning Light Treatment with Stable Sleep Timing for Individuals with Fibromyalgia: A Randomized Controlled Trial	Pain medicine (Malden, Mass.)	wrong intervention

2023	Méndez-Sánchez, L.; Clark, P.; Winzenberg, T.M.; Tugwell, P.; Correa-Burrows, P.; Costello, R.	Calcium and vitamin D for increasing bone mineral density in premenopausal women	Cochrane Database of Systematic Reviews	wrong population
2023	Poh, P.Y.S.; Sessoms, P.H.; Haluch, K.S.; Trone, D.W.	Assessing Injury Susceptibility at Marine Corps Recruit Depot, San Diego, California	Journal of strength and conditioning research	wrong population
2023	Mota Neto, J.; Mendes, A.F.; Martins, A.F.M.; Landa, A.T.; Fraga, R.O.; Souza, V.A.; Raposo, N.R.B.	Protocol of HOTFy: randomised clinical trial to hyperbaric oxygen therapy in fibromyalgia	BMJ open	wrong intervention
2023	Zhang, M.; Liang, X.; Huang, W.; Ding, S.; Li, G.; Zhang, W.; Li, C.; Zhou, Y.; Sun, J.; Li, D.	The effects of velocity-based versus percentage-based resistance training on athletic performances in sport-collegiate female basketball players	Frontiers in Physiology	wrong population
2023	Johnson, Q.R.; Scraper, J.; Lockie, R.; Orr, R.M.; Dawes, J.J.	Sex-related Differences in Functional Movement Screen Scores Among Reserve Officers' Training Corps Cadets	Military medicine	wrong population
2023	Caglayan, B.C.; Basakci Calik, B.; Gur Kabul, E.; Karasu, U.	Investigation of effectiveness of reformer pilates in individuals with fibromyalgia: A randomized controlled trial	Reumatologia Clinica	wrong intervention
2023	Franco, K.F.M.; Miyamoto, G.C.; Franco, Y.R.D.S.; Salvador, E.M.E.S.; do Nascimento, B.C.B.; Menten, L.A.; Cabral, C.M.N.	Is Pilates more effective and cost-effective than aerobic exercise in the treatment of patients with fibromyalgia syndrome? A randomized controlled trial with economic evaluation	European Journal of Pain (United Kingdom)	wrong intervention
2023	Audoux, C.R.; Estrada-Barranco, C.; Martínez-Pozas, O.; Gozalo-Pascual, R.; Montaño-Ocaña, J.; García-Jiménez, D.; Vicente de Frutos, G.; Cabezas-Yagüe, E.; Sánchez Romero, E.A.	What Concept of Manual Therapy Is More Effective to Improve Health Status in Women with Fibromyalgia Syndrome? A Study Protocol with Preliminary Results	International Journal of Environmental Research and Public Health	wrong study design

2023		Correction: Are There Interindividual Differences in Anxiety as a Result of Aerobic Exercise Training in Adults With Fibromyalgia? An Ancillary Meta-analysis of Randomized Controlled Trials	Archives of Physical Medicine and Rehabilitation	wrong study design
2023	Alptug, B.; Tüzün, E.H.; Keçeci, B.; Eker, L.	Effects of perceptive rehabilitation and mobilization methods on symptoms and disability in patients with fibromyalgia: A preliminary randomized control trial	Irish Journal of Medical Science	wrong study design
2023	Löfgren, M.; Sandström, A.; Bileviciute-Ljungar, I.; Mannerkorpi, K.; Gerdle, B.; Ernberg, M.; Fransson, P.; Kosek, E.	The effects of a 15-week physical exercise intervention on pain modulation in fibromyalgia: Increased pain-related processing within the cortico-striatal- occipital networks, but no improvement of exercise-induced hypoalgesia	Neurobiology of Pain	wrong intervention
2023	Bossenger, N.R.; Lewis, G.N.; Rice, D.A.; Shepherd, D.	The autonomic and nociceptive response to acute exercise is impaired in people with knee osteoarthritis	Neurobiology of Pain	wrong population
2023	Wade, L.; Beauchamp, M.R.; Nathan, N.; Smith, J.J.; Leahy, A.A.; Kennedy, S.G.; Boyer, J.; Bao, R.; Diallo, T.M.O.; Vidal-Conti, J.; Lubans, D.R.	Investigating the direct and indirect effects of a school-based leadership program for primary school students: Rationale and study protocol for the 'Learning to Lead' cluster randomised controlled trial	PLoS ONE	wrong population
2023	Sang, X.	EFFECTS OF FUNCTIONAL TRAINING ON THE ATHLETIC QUALITY OF AEROBICS PRACTITIONERS	Revista Brasileira de Medicina do Esporte	wrong population
2023	Huang, H.	EFFECTS OF RUNNING ON SPORTS INJURIES DURING REHABILITATION	Revista Brasileira de Medicina do Esporte	wrong population
2023	Li, J.; Liu, M.	EFFECTS OF FUNCTIONAL TRAINING ON THE PERFORMANCE OF SEDENTARY COLLEGE STUDENTS	Revista Brasileira de Medicina do Esporte	wrong population

2023	Jiang, W.	EFFECTS OF PHYSICAL CONDITIONING ON TEACHING SWIMMING SKILLS TO UNIVERSITY STUDENTS	Revista Brasileira de Medicina do Esporte	wrong population
2023	Wu, M.; Su, Z.; Wan, M.	JOINT INJURIES IN MARTIAL ARTS AND THEIR PREVENTIVE MEASURES	Revista Brasileira de Medicina do Esporte	wrong population
2023	Xiao, L.	ANALYSIS AND PREVENTIVE MEASURES FOR NON-CONTACT INJURIES IN SOCCER	Revista Brasileira de Medicina do Esporte	wrong population
2023	Huihui, X.	INJURY PREVENTION FOR YOGA PRACTICING COLLEGE STUDENTS	Revista Brasileira de Medicina do Esporte	wrong population
2023	Byrom, B.; Bessant, C.; Smeraldi, F.; Abdollahyan, M.; Bridges, Y.; Chowdhury, M.; Tahsin, A.	Deriving Meaningful Aspects of Health Related to Physical Activity in Chronic Disease: Concept Elicitation Using Machine Learning–Assisted Coding of Online Patient Conversations	Value in Health	wrong intervention
2023	Luo, K.; Zhang, W.	INVESTIGATION AND PREVENTION OF SPORTS RISK IN THE TEACHING OF TABLE TENNIS	Revista Brasileira de Medicina do Esporte	wrong population
2023	Bradshaw, B.T.; Hunt, A.W.; Ludwig, E.; Newcomb, T.L.	Dental hygiene students' matching accuracy when comparing antemortem dental radiographs and oral photographs to simulated postmortem WinID3® odontograms	Journal of forensic sciences	wrong population

2023	Veličković, Z.; Janjić, S.; Radunović, G.	Accelerometry-based assessment of balance alterations in patientswith primary fibromyalgia -Pilot cross-sectional study	International Journal of Rheumatic Diseases	wrong study design
2023	Hermankova, B.; Oreska, S.; Spiritovic, M.; Storkanova, H.; Pavelka, K.; Senolt, L.; Vencovsky, J.; Becvar, R.; Tomcik, M.	EFFECT OF AN 8-WEEK PHYSICAL THERAPY PROGRAM ON SEXUAL FUNCTION IN FEMALE PATIENTS WITH IDIOPATHIC INFLAMMATORY MYOPATHIES AND SYSTEMIC SCLEROSIS: A PILOT CONTROLLED STUDY	Clinical and Experimental Rheumatology	wrong intervention
2023	Vernerová, L.; Vokurková, M.; Oreská, S.; Špiritović, M.; Klein, M.; Kropáčková, T.; Horváthová, V.; Tomčík, M.; Ukropec, J.; Ukropcová, B.; Vencovský, J.	VITAMIN D AND THE GENE EXPRESSION OF ITS RECEPTOR (VDR) IN SKELETAL MUSCLE PLAY A ROLE IN DISEASE MANIFESTATION AND PHYSICAL FITNESS OF PATIENTS WITH MYOSITIS	Clinical and Experimental Rheumatology	wrong intervention
2023	Aggarwal, R.; Pisarczyk, K.; Leff, R.; Park, E.; Palaniswamy, K.; Long, L.	DIRECT AND INDIRECT COSTS ASSOCIATED WITH THE MANAGEMENT OF DERMATOMYOSITIS AND POLYMYOSITIS IN ADULT PATIENTS	Clinical and Experimental Rheumatology	wrong intervention
2022	Torres Vilarino, G.; Reis Coimbra, D.; Guimarães Bevilacqua, G.; Diotaiuti, P.; Falese, L.; Andrade, A.	Can different degrees of resistance training improve mood states in patients with fibromyalgia? A randomized controlled trial	Reumatismo	wrong intervention
2022	Hradetzky, E.; Ohlmeier, C.; Brinkmann, C.; Schild, M.; Galetzka, W.; Schmedt, N.; John, T.; Kaleth, D.; Gothe, H.	Epidemiology and routine care treatment of patients with hip or knee osteoarthritis and chronic lower back pain: real-world evidence from Germany	Journal of Public Health (Germany)	wrong population
2022	Jones, T.; O'Grady, K.-A.F.; Goyal, V.; Masters, I.B.; McCallum, G.; Drovandi, C.; Lung, T.; Baque, E.; Brookes, D.S.K.; Terranova, C.O.; Chang, A.B.; Trost, S.G.	Bronchiectasis - Exercise as Therapy (BREATH): rationale and study protocol for a multi-center randomized controlled trial	Trials	wrong population

2022	Scalabrini, J.C.; Schiffenbauer, A.I.; Farhadi, P.N.; Volochayev, R.; Bayat, N.; Jansen, A.; Targoff, I.N.; Miller, F.W.; Rider, L.G.	Environmental factors associated with juvenile idiopathic inflammatory myopathy clinical and serologic phenotypes	Pediatric Rheumatology	wrong intervention
2022	Pearson, J.; Coggins, J.; Derham, S.; Russell, J.; Walsh, N.E.; Lenguerrand, E.; Palmer, S.; Cramp, F.	A feasibility randomised controlled trial of a Fibromyalgia Self-management Programme for adults in a community setting with a nested qualitative study (FALCON)	BMC Musculoskeletal Disorders	wrong intervention
2022	Hurrell, A.; Sparkes, J.; Duhig, K.; Seed, P.T.; Myers, J.; Battersby, C.; Clark, K.; Green, M.; Hunter, R.M.; Shennan, A.H.; Chappell, L.C.; Webster, L.	Placental growth fActor Repeat sampling for Reduction of adverse perinatal Outcomes in women with suspecTed pre-eclampsia: study protocol for a randomised controlled trial (PARROT-2)	Trials	wrong population
2022	dos Santos, K.V.G.; da Silva Leal, K.C.; de Melo Alves Silva, L.C.; de Medeiros, K.S.; Feijão, A.R.; de Oliveira, M.C.; Dantas, D.V.; Dantas, R.A.N.	Music-induced analgesia for adults and older adults during femoral arterial sheath removal after cardiac catheterization: a randomized clinical trial protocol	BMC Complementary Medicine and Therapies	wrong population
2022	Yang, G.-Y.; Hunter, J.; Bu, F.-L.; Hao, W.-L.; Zhang, H.; Wayne, P.M.; Liu, J.-P.	Determining the safety and effectiveness of Tai Chi: a critical overview of 210 systematic reviews of controlled clinical trials	Systematic Reviews	wrong study design
2022	Kashikar-Zuck, S.; Barnett, K.A.; Williams, S.E.; Pfeiffer, M.; Thomas, S.; Beasley, K.; Chamberlin, L.A.; Mundo, K.; Ittenbach, R.F.; Peugh, J.; Gibler, R.C.; Lynch-Jordan, A.; Ting, T.V.; Gadd, B.; Taylor, J.; Goldstein-Leever, A.; Connelly, M.; Logan, D.E.; Williams, A.; Wakefield, E.O.; Myer, G.D.	FIT Teens RCT for juvenile fibromyalgia: Protocol adaptations in response to the COVID 19 pandemic	Contemporary Clinical Trials Communications	wrong intervention

2022	Landman, T.R.J.; Uthman, L.; Hofmans, I.A.H.; Schoon, Y.; de Leeuw, F.-E.; Thijssen, D.H.J.	Attenuated inflammatory profile following single and repeated handgrip exercise and remote ischemic preconditioning in patients with cerebral small vessel disease	Frontiers in Physiology	wrong population
2022	Serrat, M.; Ferrés, S.; Auer, W.; Almirall, M.; Lluch, E.; D'Amico, F.; Maes, M.; Lorente, S.; Navarrete, J.; Montero-Marín, J.; Neblett, R.; Nijs, J.; Borràs, X.; Luciano, J.V.; Feliu-Soler, A.	Effectiveness, cost-utility and physiological underpinnings of the FIBROWALK multicomponent therapy in online and outdoor format in individuals with fibromyalgia: Study protocol of a randomized, controlled trial (On&Out study)	Frontiers in Physiology	wrong intervention
2022	Kabir, T.; Lee, C.-T.; Chen, L.; Jiang, X.; Shams, S.	A comprehensive artificial intelligence framework for dental diagnosis and charting	BMC oral health	wrong population
2022	Demoulin, C.; Labory, C.; Marcon, C.; Micoulau, J.R.; Dardenne, N.; Vanderthommen, M.; Kaux, J.-F.	Feasibility and Acceptability of a Home-Based Sensory Perception Training Game for Patients with Fibromyalgia: A Pilot Study	Games for health journal	wrong study design
2022	Menten, L.A.; Franco, K.F.M.; Franco, Y.R.S.; Miyamoto, G.C.; Reis, F.J.J.; Cabral, C.M.N.	Do patients with fibromyalgia have body image and tactile acuity distortion?	Pain Practice	wrong study design
2022	Schöpper, M.	Therapeutic application of Qigong/Taiji in the syndrome of fibromyalgia as a noninvasive nonpharmcological treatment	Chinesische Medizin	wrong intervention
2022	Serrat, M.; Albajes, K.; Navarrete, J.; Almirall, M.; Lluch Girbés, E.; Neblett, R.; Luciano, J.V.; Moix, J.; Feliu-Soler, A.	Effectiveness of two video-based multicomponent treatments for fibromyalgia: The added value of cognitive restructuring and mindfulness in a three-arm randomised controlled trial	Behaviour Research and Therapy	wrong intervention
2022	Xu, D.; Wu, H.; Ruan, H.; Yuan, C.; Gao, J.; Guo, M.	Effects of Yoga Intervention on Functional Movement Patterns and Mindfulness in Collegiate Athletes: A Quasi-Experimental Study	International Journal of Environmental Research and Public Health	wrong population

2022	Lichtenstein, A.; Tiosano, S.; Comaneshter, D.; Cohen, A.D.; Amital, H.	A case-control analysis of the associations between Fibromyalgia Syndrome and Osteoporosis	Israel Medical Association Journal	wrong study design
2022	Ramasawmy, P.; Khalid, S.; Petzke, F.; Antal, A.	Pain reduction in fibromyalgia syndrome through pairing transcranial direct current stimulation and mindfulness meditation: A randomized, double-blinded, sham-controlled pilot clinical trial	Frontiers in Medicine	wrong intervention
2022	Hinchado, M.D.; Otero, E.; Navarro, M.D.C.; Martín-Cordero, L.; Gálvez, I.; Ortega, E.	Influence of Codiagnosis of Chronic Fatigue Syndrome and Habitual Physical Exercise on the Psychological Status and Quality of Life of Patients with Fibromyalgia	Journal of Clinical Medicine	wrong outcome
2022	Zając, B.; Mika, A.; Gaj, P.K.; Ambroży, T.	Does Cycling Training Reduce Quality of Functional Movement Motor Patterns and Dynamic Postural Control in Adolescent Cyclists? A Pilot Study	International Journal of Environmental Research and Public Health	wrong study design
2022	Hafsteinsson Östenberg, A.; Enberg, A.; Pojskic, H.; Gilic, B.; Sekulic, D.; Alricsson, M.	Association between Physical Fitness, Physical Activity Level and Sense of Coherence in Swedish Adolescents; An Analysis of Age and Sex Differences	International Journal of Environmental Research and Public Health	wrong population
2022	Brown, I.M.; Cancado, D.K.; Chase, B.L.; Nakachi, E.H.; Reece, J.D.	The acute effects of self-myofascial release on fundamental physical activity movement patterns	Obesity Reviews	wrong intervention
2022	Adler, S.; Lutz, J.; Napadow, V.; Hare, B.D.; Gupta, A.; Lakhan, S.	A BASKET TRIAL FOR ACCELERATED DISCOVERY OF NOVEL NEUROSCIENCEBASED DIGITAL TREATMENTS FOR CHRONIC PAIN	Innovations in Clinical Neuroscience	wrong study design
2022	Puthupparmbil, J.J.; Sofat, N.	A RARE CASE OF COSTOVERTEBRAL ARTHRITIS IN SLE Afzal Latheef1	Rheumatology Advances in Practice	wrong population

2022	Shan, Z.Y.; Mohamed, A.Z.; Andersen, T.; Rendall, S.; Kwiatek, R.A.; Fante, P.D.; Calhoun, V.D.; Bhuta, S.; Lagopoulos, J.	Multimodal MRI of myalgic encephalomyelitis/chronic fatigue syndrome: A cross-sectional neuroimaging study toward its neuropathophysiology and diagnosis	Frontiers in Neurology	wrong population
2022	Joschtel, B.; Gomersall, S.R.; Tweedy, S.; Petsky, H.; Chang, A.B.; Trost, S.G.	Effects of a therapeutic exercise program in children with non-cystic fibrosis bronchiectasis: A pilot randomized controlled trial	Frontiers in Pediatrics	wrong population
2022	Kaushik, D.; Shah, P.K.; Mukherjee, N.; Ji, N.; Dursun, F.; Kumar, A.P.; Thompson, I.M.; Mansour, A.M.; Jha, R.; Yang, X.; Wang, H.; Darby, N.; Ricardo Rivero, J.; Svatek, R.S.; Liss, M.A.	Effects of yoga in men with prostate cancer on quality of life and immune response: a pilot randomized controlled trial	Prostate Cancer and Prostatic Diseases	wrong population
2022	Mehta, P.; Basu, A.; Ahmed, S.	Effectiveness and adverse effects of the use of mirtazapine as compared to duloxetine for fibromyalgia: real-life data from a retrospective cohort	Rheumatology International	wrong study design
2022	Kelley, G.A.; Kelley, K.S.; Callahan, L.F.	Are There Interindividual Differences in Anxiety as a Result of Aerobic Exercise Training in Adults With Fibromyalgia? An Ancillary Meta-analysis of Randomized Controlled Trials	Archives of Physical Medicine and Rehabilitation	wrong intervention
2022	Kolak, E.; Ardıç, F.; Fındıkoğlu, G.	Effects of different types of exercises on pain, quality of life, depression, and body composition in women with fibromyalgia: A three-arm, parallel-group, randomized trial	Archives of Rheumatology	wrong intervention
2022	Mikkonen, J.; Leinonen, V.; Kaski, D.; Hartvigsen, J.; Luomajoki, H.; Selander, T.; Airaksinen, O.	Postural sway does not differentiate individuals with chronic low back pain, single and multisite chronic musculoskeletal pain, or pain-free controls: a cross-sectional study of 229 subjects	Spine Journal	wrong population

2022	Kim, B.-J.; Park, A.-L.; Hwang, M.-S.; Heo, I.; Park, S.-Y.; Cho, J.-H.; Kim, K.-W.; Lee, J.-H.; Ha, I.-H.; Park, K.-S.; Hwang, E.-H.; Shin, B.-C.	Comparative Effectiveness and Safety of Concomitant Treatment with Chuna Manual Therapy and Usual Care for Whiplash Injuries: A Multicenter Randomized Controlled Trial	International Journal of Environmental Research and Public Health	wrong population
2022	Costa, A.R.; Freire, A.; Parraca, J.A.; Silva, V.; Tomas-Carus, P.; Villafaina, S.	Heart Rate Variability and Salivary Biomarkers Differences between Fibromyalgia and Healthy Participants after an Exercise Fatigue Protocol: An Experimental Study	Diagnostics	wrong intervention
2022	Brandt, T.; Schmidt, A.; Schinköthe, T.; Heinz, E.; Klaaßen, Y.; Limbara, S.; Mörsdorf, M.	MedXFit—Effects of 6 months CrossFit® in sedentary and inactive employees: A prospective, controlled, longitudinal, intervention study	Health Science Reports	wrong population
2022	Ong, A.D.; Moskowitz, J.T.; Wethington, E.; Addington, E.L.; Sanni, M.; Goktas, S.; Sluys, E.; Swong, S.; Kim, P.; Reid, M.C.	Lessons in Affect Regulation to Keep Stress and Pain UndeR control (LARKSPUR): Design of a randomized controlled trial to increase positive affect in middle-aged and older adults with fibromyalgia	Contemporary Clinical Trials	wrong intervention
2022	Farias Teixeira, M.E.; Bonjorno, L.P.; Menezes, P.I.S.O.; Gigante, S.L.G.; Dib, S.M.; Oliveira, F.R.; Silva, L.M.; Petean, F.C.; Moraes, D.A.; Luppino-Assad, R.; Oliveira, R.; Almeida, S.C.L.; Oliveira, M.C.; Louzada-Junior, P.; Oliveira, R.D.R.	Diagnostic Agreement Among General Practitioners, Residents, and Senior Rheumatologists for Rheumatic Diseases	Journal of Clinical Rheumatology	wrong study design
2022	Ramasawmy, P.; Khalid, S.; Petzke, F.; Antal, A.	MINDFULLY STIMULATING THE BRAIN: A RANDOMIZED, SHAM-CONTROLLED AND DOUBLE-BLINDED PILOT CLINICAL TRIAL PAIRING TRANSCRANIAL DIRECT CURRENT STIMULATION AND MINDFULNESS MEDITATION IN FIBROMYALGIA	Pain Practice	wrong intervention
2022	Lavarello, C.; Alongi, A.; Mori, B.; Ronchetti, A.; Nobili, L.; Chiarella, L.; Pescio, E.; Ravelli, A.; Gattorno, M.; Malattia, C.	A novel multidimensional questionnaire to monitor juvenile fibromyalgia syndrome and identify factors influencing the disease course	Pediatric Rheumatology	wrong intervention

2022	Dai, Y.; Rosenbluth, M.; Gendreau, M.; Vega, N.; Ghalib, Z.; Kraus, A.; Keefe, B.	Clinical Impact of a Digital Behavioral Therapy for Fibromyalgia Management in a Decentralized Trial	Arthritis and Rheumatology	wrong intervention
2022	Post, A.A.; Dailey, D.L.; Bayman, E.O.; Chimenti, R.L.; Costigan, M.; Franck, C.; Huff, T.; Johnson, E.; Koepp, M.; Lafontant, D.-E.; McCabe, M.E.; Neill-Hudson, T.; Vance, C.G.T.; Van Gorp, B.; Zimmerman, B.M.; Ecklund, D.; Crofford, L.J.; Sluka, K.A.	The Fibromyalgia Transcutaneous Electrical Nerve Stimulation in Physical Therapy Study (FM-TIPS) Protocol: A Multisite Embedded Pragmatic Trial	Physical therapy	wrong intervention
2022	Arroyo-Fernández, R.; Avendaño-Coy, J.; Velasco-Velasco, R.; Palomo-Carrión, R.; Bravo-Esteban, E.; Ferri-Morales, A.	Effectiveness of Transcranial Direct Current Stimulation Combined With Exercising in People With Fibromyalgia: A Randomized Sham-Controlled Clinical Trial	Archives of Physical Medicine and Rehabilitation	wrong intervention
2022	Salgado, A.S.I.; Takemoto, M.H.; de Souza, C.F.T.C.; Salm, D.C.; da Rosa, D.; Cardoso, G.C.; Ludtke, D.D.; Soares, S.F.C.; Ferreira, J.K.; Dutra, A.R.; Szeremeta, Y.C.; Mazzardo, G.; Mayra, J.; Sheffer, D.D.L.; Caumo, W.; Bittencourt, E.B.; Schleip, R.; Latini, A.; Bobinski, F.; Martins, D.F.	Gentle Touch Therapy, Pain Relief and Neuroplasticity at Baseline in Fibromyalgia Syndrome: A Randomized, Multicenter Trial with Six-Month Follow-Up	Journal of Clinical Medicine	wrong intervention
2022	Ueberall, M.A.; Horlemann, J.; Schuermann, N.; Kalaba, M.; Ware, M.A.	Effectiveness and Tolerability of Dronabinol Use in Patients with Chronic Pain: A Retrospective Analysis of 12-Week Open-Label Real-World Data Provided by the German Pain e-Registry	Pain Medicine (United States)	wrong intervention
2022	Buxton, J.D.; Prins, P.J.; Miller, M.G.; Moreno, A.; Welton, G.L.; Atwell, A.D.; Talampas, T.R.; Elsey, G.E.	The Effects of a Novel Quadrupedal Movement Training Program on Functional Movement, Range of Motion, Muscular Strength, and Endurance	Journal of strength and conditioning research	wrong intervention

2022	Shtyrov, Y.; Filippova, M.; Perikova, E.; Kirsanov, A.; Shcherbakova, O.; Blagovechtchenski, E.	Explicit encoding vs. fast mapping of novel spoken words: Electrophysiological and behavioural evidence of diverging mechanisms	Neuropsychologia	wrong population
2022	Morgan, P.J.; Rayward, A.T.; Young, M.D.; Pollock, E.R.; Eather, N.; Barnes, A.T.; Kennedy, S.-L.; Saunders, K.L.; Drew, R.J.; Lubans, D.R.	Establishing Effectiveness of a Community-based, Physical Activity Program for Fathers and Daughters: A Randomized Controlled Trial	Annals of behavioral medicine : a publication of the Society of Behavioral Medicine	wrong population
2022	Fabry, V.; Mamalet, F.; Laforet, A.; Capelle, M.; Acket, B.; Sengenes, C.; Cintas, P.; Faruch-Bilfeld, M.	A deep learning tool without muscle-by-muscle grading to differentiate myositis from facio-scapulo-humeral dystrophy using MRI	Diagnostic and Interventional Imaging	wrong intervention
2022	Schmidt, L.J.; Rieger, O.; Neznansky, M.; Hackelöer, M.; Dröge, L.A.; Henrich, W.; Higgins, D.; Verlohren, S.	A machine-learning–based algorithm improves prediction of preeclampsia-associated adverse outcomes	American Journal of Obstetrics and Gynecology	wrong population
2022	Dear, B.F.; Scott, A.J.; Fogliati, R.; Gandy, M.; Karin, E.; Dudeney, J.; Nielssen, O.; Mcdonald, S.; Heriseanu, A.I.; Bisby, M.A.; Sharpe, L.; Jones, M.P.; Ali, S.; Titov, N.	The Chronic Conditions Course: A Randomised Controlled Trial of an Internet-Delivered Transdiagnostic Psychological Intervention for People with Chronic Health Conditions	Psychotherapy and Psychosomatics	wrong population
2022	Chawla, G.; Azharuddin, M.; Ahmad, I.; Hussain, M.E.	Effect of Whole-body Vibration on Depression, Anxiety, Stress, and Quality of Life in College Students: A Randomized Controlled Trial	Oman Medical Journal	wrong population
2022	Edinoff, A.N.; Fort, J.M.; Singh, C.; Wagner, S.E.; Rodriguez, J.R.; Johnson, C.A.; Cornett, E.M.; Murnane, K.S.; Kaye, A.M.; Kaye, A.D.	Alternative Options for Complex, Recurrent Pain States Using Cannabinoids, Psilocybin, and Ketamine: A Narrative Review of Clinical Evidence	Neurology International	wrong population

2022	Huang, X.; Liu, H.	Criterion Validity of Functional Movement Screen as a Predictor of Sports Injury Risk in Chinese Police Staff	International Journal of Environmental Research and Public Health	wrong population
2022	Burgess, H.; Bahl, S.; Wilensky, K.; Spence, E.; Jouppi, R.; Rizvydeen, M.; Goldstein, C.; Williams, D.; Kim, M.; Burns, J.	A 4-WEEK SLEEP INTERVENTION THAT ADVANCES AND STABILIZES SLEEP TIMING LEADS TO MEANINGFUL IMPROVEMENTS IN PAIN AND PHYSICAL FUNCTION IN PEOPLE WITH FIBROMYALGIA	Sleep	wrong intervention
2022	Zangi, H.A.; Haugmark, T.; Provan, S.A.; Hagen, K.B.	HIGH SYMPTOM BURDEN and LIMITED CHANGES in FIBROMYALGIA PATIENTS' HEALTH STATUS TWO YEARS after PARTICIPATION in A MULTICOMPONENT REHABILITATION PROGRAMME	Annals of the Rheumatic Diseases	wrong intervention
2022	Vernerová, L.; Vokurková, M.; Oreska, S.; Špiritović, M.; Klein, M.; Kropackova, T.; Horvathova, V.; Tomčík, M.; Ukropec, J.; Ukropcová, B.; Vencovský, J.	VITAMIN D and ITS RECEPTOR (VDR) GENE EXPRESSION in SKELETAL MUSCLE ASSOCIATE with DISEASE and MUSCLE FUNCTION PARAMETERS in IDIOPATHIC INFLAMMATORY MYOPATHIES	Annals of the Rheumatic Diseases	wrong intervention
2022	Gulsen, C.; Soke, F.; Eldemir, K.; Apaydin, Y.; Ozkul, C.; Guclu-Gunduz, A.; Akcali, D.T.	Effect of fully immersive virtual reality treatment combined with exercise in fibromyalgia patients: a randomized controlled trial	Assistive technology : the official journal of RESNA	wrong intervention
2022	Wang, G.; Zi, Y.; Li, B.; Su, S.; Sun, L.; Wang, F.; Ren, C.; Liu, Y.	The Effect of Physical Exercise on Fundamental Movement Skills and Physical Fitness among Preschool Children: Study Protocol for a Cluster-Randomized Controlled Trial	International Journal of Environmental Research and Public Health	wrong population
2022	Marquina-Márquez, A.; Olry-De Labry-Lima, A.; Bermúdez-Tamayo, C.; Ferrer López, I.; Marcos-Marcos, J.	Identifying barriers and enablers for benzodiazepine (de)prescription: a qualitative study with patients and healthcare professionals	Anales del Sistema Sanitario de Navarra	wrong population

2022	Bayman, E.O.; Dailey, D.; Ecklund, D.; Johnson, E.; Vance, C.; Gorp, B.V.; Lafontant, D.-E.; McCabe, M.; Zimmerman, B.; Koepp, M.; Costigan, M.; Chimenti, R.; Spencer, M.; Post, A.; Huff, T.; Archer, K.; Neill-Hudson, T.; Peters, R.; Nye, G.; Franck, C.; Crofford, L.; Sluka, K.	Impact of COVID-19 on the Patient Enrollment for a Pragmatic, Cluster Randomized Clinical Trial for Fibromyalgia	Journal of Pain	wrong population
2022	Khataei, T.; Harding, A.M.; Snyder, P.M.; Sluka, K.A.; Pierce, G.L.; Benson, C.J.	ASIC3 Plays Protective Role in Delayed-onset Muscle Soreness via Muscle Acid Sensation During Maximal Exercise	Journal of Pain	wrong population
2022	Engel, A.; Broderick, C.; Hardy, L.; Ward, R.; van Doorn, N.; Kwai, N.; Parmenter, B.	Effect of a Fundamental Motor Skills Intervention on Fundamental Motor Skill and Physical Activity in a Preschool Setting: A Cluster Randomized Controlled Trial	Pediatric exercise science	wrong population
2022	Ghadiri, F.; Bahmani, M.; Paulson, S.; Sadeghi, H.	Effects of fundamental movement skills based dual-task and dance training on single- and dual-task walking performance in older women with dementia	Geriatric nursing (New York, N.Y.)	wrong population
2022	Pearson, J.; Coggins, J.; Derham, S.; Russell, J.; Walsh, N.; Lenguerrand, E.; Palmer, S.; Cramp, F.	A FEASIBILITY RANDOMISED CONTROLLED TRIAL OF A FIBROMYALGIA SELF-MANAGEMENT PROGRAMME IN A COMMUNITY SETTING WITH A NESTED QUALITATIVE STUDY	Rheumatology (United Kingdom)	wrong intervention
2022	Notch, D.; Krashin, D.; Murinova, N.	Out of Sight and Out of Mind: The Nocebo Effect; An Unmet Educational Need in Academic Medical Centers	Neurology	wrong population
2022	Bagagiolo, D.; Debora, R.; Borrelli, F.	Efficacy and safety of osteopathic manipulative treatment: An overview of systematic reviews	BMJ Open	wrong study design
2022	Dunn, T.J.; Dimolareva, M.	The effect of mindfulness-based interventions on immunity-related biomarkers: a comprehensive meta-analysis of randomised controlled trials	Clinical Psychology Review	wrong study design
2022	Gucmen, B.; Kocyigit, B.F.; Nacitarhan, V.; Berk, E.; Koca, T.T.; Akyol, A.	The relationship between cervical proprioception and balance in patients with fibromyalgia syndrome	Rheumatology International	wrong intervention

2022	Schamne, J.C.; Ressetti, J.C.; Bertuzzi, R.; Okuno, N.M.; Lima-Silva, A.E.	Acute Caffeine Intake Reduces Perceived Exertion But Not Muscle Pain during Moderate Intensity Cycling Exercise in Women with Fibromyalgia	Journal of the American Nutrition Association	wrong intervention
2022	Zhang, Y.; Xue, R.; Tong, Z.; Yin, M.; Yu, Y.; Ye, J.; Xu, J.; Mo, W.	The Efficacy of Manipulation with Distension Arthrography to Treat Adhesive Capsulitis: A Multicenter, Randomized, Single-Blind, Controlled Trial	BioMed Research International	wrong population
2022	Scolaro, L.L.; Cremoneze, I.Z.; de Moura-Costa, G.F.	The Pharmacy of the Specialized Component of Pharmaceutical Policy of the 9th Regional Health Department of the State of Paraná and the Treatment of Ankylosing Spondylitis	Brazilian Journal of Pharmaceutical Sciences	conference abstract
2022	Wang, J.	INFLUENCE OF PHYSICAL TRAINING ON THE PHYSICAL QUALITY OF UNIVERSITY STUDENTS	Revista Brasileira de Medicina do Esporte	wrong population
2022	Kitt-Lewis, E.; Adam, M.; Berish, D.	The roles and experiences of family members who care for a person with substance or opioid use disorder	Journal of Substance Use	wrong population
2022	Kim, J.H.; Hong, Y.J.; Lee, G.	Efficacy of a Lower Back Intensive Rehabilitation Program in Occupational Injury Patients and Characteristics of Care: A Retrospective Cohort Study	Medical Science Monitor	wrong population
2022	Bihi, T.G.; Kusakana, K.	An Intelligent Quality Management System for Flexible Manufacturing Systems: Case of Circuit Breakers Assembly System with Interchangeable Tools/Stations	NeuroQuantology	wrong intervention
2022	Nasonova, T.I.; Parfenova, E.V.	Comorbidities and therapy for chronic non-specific low back pain and fibromyalgia	Nevrologiya, Neiropsikhiatriya, Psikhosomatika	wrong intervention
2022	Gomez-Arguelles, J.M.; Caceres, O.; Blanco, M.; Maestu, C.; Martin, F.	Improvement of digestive symptoms in fibromyalgia patients following a diet modification according to histamine release test - an observational study	Reumatologia	wrong study design

2022	Lynch-Jordan, A.M.; Connelly, M.; Guite, J.W.; King, C.; Goldstein-Leever, A.; Logan, D.E.; Nelson, S.; Stinson, J.N.; Ting, T.V.; Wakefield, E.O.; Williams, A.E.; Williams, S.E.; Kashikar-Zuck, S.	Clinical Characterization of Juvenile Fibromyalgia in a Multicenter Cohort of Adolescents Enrolled in a Randomized Clinical Trial	Arthritis Care and Research	wrong intervention
2022	Yoo, S.-A.; Kim, C.-Y.; Kim, H.-D.; Kim, S.-W.	Effects of progressive muscle relaxation therapy with home exercise on pain, fatigue, and stress in subjects with fibromyalgia syndrome: A pilot randomized controlled trial	Journal of Back and Musculoskeletal Rehabilitation	wrong intervention
2022	Paolucci, T.; Agostini, F.; Mangone, M.; Torquati, A.; Scienza, S.; Sorgi, M.L.; Pellicano, G.R.; Lai, C.; Bernetti, A.; Paoloni, M.	Effects of a motor imagery-based exercise protocol on chronic pain and emotional distress in patients with fibromyalgia syndrome: A pilot study	Journal of Back and Musculoskeletal Rehabilitation	wrong study design
2022	De Lorena, S.B.; Duarte, A.L.B.P.; Bredemeier, M.; Fernandes, V.M.; Pimentel, E.A.S.; Marques, C.D.L.; Ranzolin, A.	Effects of a physical self-care support program for patients with fibromyalgia: A randomized controlled trial	Journal of Back and Musculoskeletal Rehabilitation	wrong intervention
2022	Fabio, G.	Why are Patients Associations so important both for doctors and patients?	Clinical and Experimental Rheumatology	wrong study design
2022	Férez, I.B.; Férez, J.B.; Escudero, M.G.; Vera, F.F.; Fito, T.S.; García, E.O.	Changes in postural control after physiotherapy and pain relief in patients with fibromyalgia	Clinical and Experimental Rheumatology	wrong intervention
2022	Lin, X.; Zhang, K.; Meng, T.	AKAP12 and RNF11 as Diagnostic Markers of Fibromyalgia and Their Correlation with Immune Infiltration	Oxidative medicine and cellular longevity	wrong intervention
2021	Gali, J.C.; Fadel, G.W.; Marques, M.F.; Almeida, T.A.; Filho, J.C.G.; Faria, F.A.S.	The New Injuries' Risk After Acl Reconstruction Might Be Reduced With Functional Training	Acta Ortopedica Brasileira	wrong population

2021	Duan, L.	Empirical analysis on the reduction of sports injury by functional movement screening method under biological image data	Revista Brasileira de Medicina do Esporte	wrong population
2019	Srinivasan, A.; Tadros, M.; Nelson, I.; Zimmerman, M.; Haynes, S.; Ducksbury, S.; Naqvi, H.	FOUNDATION MENTORSHIP SCHEME PILOT PROGRAMME: IS NEAR-PEER MENTORSHIP AN EFFECTIVE WAY TO LOOK AFTER INCOMING FY1S?	BMJ Leader	wrong population
2017	Ataoglu, S.; Ankarali, H.; Ankarali, S.; Ataoglu, B.B.; Ölmez, S.B.	The Comparison of Rheumatic Patients in Terms of Quality of Life	Psychiatry and Clinical Psychopharmacology	wrong study design
2011	Thomason, P.; Baker, R.; Dodd, K.; Taylor, N.; Selber, P.; Wolfe, R.; Graham, H.K.	Single-event multilevel surgery in children with spastic diplegia: A pilot randomized controlled trial	Journal of Bone and Joint Surgery	wrong population
2022	Boggiss, Érika Almeida; Rosário, Rhaynara Coelho; de Lima, Rosana Aparecida; Silva, Paula Aparecida; Moreira, Rosa Maria; da Silva, Karol Priscila; de Farias, Caroline Lima; Dos Santos, Vanessa de Queiroz; Simões, Rodrigo Polaquini; Santos, Adriana Teresa Silva; Terra, Andréia Maria Silva Vilela	Pulsed Laser Acupuncture in the Treatment of Pain and Heart Rate Variability in Fibromyalgia Patients: A Pilot Randomized Clinical Trial.	Journal of lasers in medical sciences	wrong intervention
2023	Scaturro, Dalila; Vitagliani, Fabio; Signa, Gabriele; Tomasello, Sofia; Tumminelli, Luigi Giuseppe; Picelli, Alessandro; Smania, Nicola; Letizia Mauro, Giulia	Neck Pain in Fibromyalgia: Treatment with Exercise and Mesotherapy.	Biomedicines	wrong intervention

2023	Shrestha, Diggaj; Sharma, Sunita	Letter to the editor on, "Comparison of the effectiveness of dry needling and high-intensity laser therapy in the treatment of myofascial pain syndrome: a randomized single-blind controlled study".	Lasers in medical science	wrong study design
2023	Dos Santos, Jousielle Márcia; Taiar, Redha; Ribeiro, Vanessa Gonçalves César; da Silva Lage, Vanessa Kelly; Scheidt Figueiredo, Pedro Henrique; Costa, Henrique Silveira; Pereira Lima, Vanessa; Sañudo, Borja; Bernardo-Filho, Mário; Sá-Caputo, Danúbia da Cunha de; Dias Peixoto, Marco Fabrício; Mendonça, Vanessa Amaral; Rapin, Amandine; Lacerda, Ana Cristina Rodrigues	Whole-Body Vibration Training on Oxidative Stress Markers, Irisin Levels, and Body Composition in Women with Fibromyalgia: A Randomized Controlled Trial.	Bioengineering (Basel, Switzerland)	wrong intervention
2023	Alkhathami, Khalid; Alshehre, Yousef; Brizzolara, Kelli; Weber, Mark; Wang-Price, Sharon	Effectiveness of Spinal Stabilization Exercises on Movement Performance in Adults with Chronic Low Back Pain.	International journal of sports physical therapy	wrong population
2023	Cheng, Ying-Chih; Hsiao, Chia-Ying; Su, Min-I.; Chiu, Chih-Chiang; Huang, Yu-Chen; Huang, Wei-Lieh	Treating fibromyalgia with electrical neuromodulation: A systematic review and meta-analysis.	Clinical neurophysiology : official journal of the International Federation of Clinical Neurophysiology	wrong study design
2023	Moreno-Ligero, Marta; Moral-Munoz, Jose A.; Salazar, Alejandro; Failde, Inmaculada	mHealth Intervention for Improving Pain, Quality of Life, and Functional Disability in Patients With Chronic Pain: Systematic Review.	JMIR mHealth and uHealth	wrong study design

2023	Loreti, Eduardo Henrique; Freire, Ariana Mendes; Alexandre da Silva, Alisson; Kakuta, Elaine; Martins Neto, Ubiratan Ribeiro; Konkiewitz, Elisabete Castelon	Effects of Anodal Transcranial Direct Current Stimulation on the Primary Motor Cortex in Women With Fibromyalgia: A Randomized, Triple-Blind Clinical Trial.	Neuromodulation : journal of the International Neuromodulation Society	wrong intervention
2022	Kohl, Jan; Brame, Judith; Hauff, Pascal; Wurst, Ramona; Sehlbrede, Matthias; Fichtner, Urs Alexander; Armbruster, Christoph; Tinsel, Iris; Maiwald, Phillip; Farin-Glattacker, Erik; Fuchs, Reinhard; Gollhofer, Albert; König, Daniel	Effects of a Web-Based Weight Loss Program on the Healthy Eating Index-NVS in Adults with Overweight or Obesity and the Association with Dietary, Anthropometric and Cardiometabolic Variables: A Randomized Controlled Clinical Trial.	Nutrients	wrong population
2023	Caglayan, Berna Cagla; Basakci Calik, Bilge; Gur Kabul, Elif; Karasu, Ugur	Investigation of effectiveness of reformer pilates in individuals with fibromyalgia: A randomized controlled trial.	Reumatologia clinica	wrong intervention
2023	Calles Plata, Irene; Ortiz-Rubio, Araceli; Torres Sánchez, Irene; Cabrera Martos, Irene; Calvache Mateo, Andrés; Heredia-Ciuró, Alejandro; Valenza, Marie Carmen	Effectiveness of aquatic therapy on sleep in persons with fibromyalgia. A meta-analysis.	Sleep medicine	wrong study design
2022	Vrouva, Sotiria; Sopidou, Varvara; Koutsioumpa, Evangelia; Chanopoulos, Konstantinos; Nikolopoulou, Alexandra; Papatsimpas, Vasileios; Koumantakis, George A.	Can Exercise Affect the Pain Characteristics in Patients with Fibromyalgia? A Randomized Controlled Trial.	Healthcare (Basel, Switzerland)	wrong intervention
2022	Suzuki, Kenta; Mizoguchi, Yasuaki; Kimura, Fumihiko; Sawada, Yutaka; Akasaka, Kiyokazu	Efficacy of Injury Prevention Using Functional Movement Screen Training in High-School Baseball Players: Secondary Outcomes of a Randomized Controlled Trial.	Healthcare (Basel, Switzerland)	wrong population

2022	Hernando-Garijo, Ignacio; Medrano-de-la-Fuente, Ricardo; Jiménez-Del-Barrio, Sandra; Mingo-Gómez, María Teresa; Hernández-Lázaro, Héctor; Lahuerta-Martin, Silvia; Ceballos-Laita, Luis	Effects of a Telerehabilitation Program in Women with Fibromyalgia at 6-Month Follow-Up: Secondary Analysis of a Randomized Clinical Trial.	Biomedicines	wrong intervention
2022	Ahi, Emine Dundar; Sirzai, Hulya	Comparison of the effectiveness of dry needling and high-intensity laser therapy in the treatment of myofascial pain syndrome: a randomized single-blind controlled study.	Lasers in medical science	wrong intervention
2022	Jimenez-Garcia, John A.; Miller, Matthew B.; DeMont, Richard G.	Effects of Multicomponent Injury Prevention Programs on Children and Adolescents' Fundamental Movement Skills: A Systematic Review With Meta-Analyses.	American journal of health promotion : AJHP	wrong population
2022	Oppici, Luca; Stell, Frederike Marie; Utesch, Till; Woods, Carl T.; Foweather, Lawrence; Rudd, James R.	A Skill Acquisition Perspective on the Impact of Exergaming Technology on Foundational Movement Skill Development in Children 3-12 Years: A Systematic Review and Meta-analysis.	Sports medicine - open	wrong population
2022	Christensen, Steffan Wittrup McPhee; Almsborg M, Heidi; Vain M, Thomas Søgaard; Vaegter, Henrik Bjarke	The Effect of Virtual Reality on Cold Pain Sensitivity in Patients with Fibromyalgia and Pain-Free Individuals: A Randomized Crossover Study.	Games for health journal	wrong intervention
2022	Zhang, Kang-Da; Wang, Lin-Yu; Zhang, Zhi-Hua; Zhang, Dan-Xu; Lin, Xiao-Wen; Meng, Tao; Qi, Feng	Effect of Exercise Interventions on Health-Related Quality of Life in Patients with Fibromyalgia Syndrome: A Systematic Review and Network Meta-Analysis.	Journal of pain research	wrong study design
2022	Cavaggioni, Luca; Gilardini, Luisa; Redaelli, Gabriella; Croci, Marina; Cancello, Raffaella; Capodaglio, Paolo; Bruno, Amalia; Bertoli, Simona	A Pilot Study on Attentional Focus in Prescribing Physical Exercise in Outpatients with Obesity.	Healthcare (Basel, Switzerland)	wrong population

2023	Navarro-Ledesma, Santiago; Carroll, James; Burton, Patricia; Ana, González-Muñoz	Short-Term Effects of Whole-Body Photobiomodulation on Pain, Quality of Life and Psychological Factors in a Population Suffering from Fibromyalgia: A Triple-Blinded Randomised Clinical Trial.	Pain and therapy	wrong intervention
2022	Navarro-Ledesma, Santiago; Carroll, James; González-Muñoz, Ana; Pruimboom, Leo; Burton, Patricia	Changes in Circadian Variations in Blood Pressure, Pain Pressure Threshold and the Elasticity of Tissue after a Whole-Body Photobiomodulation Treatment in Patients with Fibromyalgia: A Tripled-Blinded Randomized Clinical Trial.	Biomedicines	wrong intervention
2022	Vilarino, Guilherme Torres; Branco, Joaquim Henrique Lorenzetti; de Souza, Loiane Cristina; Andrade, Alexandro	Effects of resistance training on the physical symptoms and functional capacity of patients with fibromyalgia: a systematic review and meta-analysis of randomized clinical trials.	Irish journal of medical science	wrong intervention
2022	Kjellberg, Anders; Abdel-Halim, Lina; Hassler, Adrian; El Gharbi, Sara; Al-Ezerjawi, Sarah; Boström, Emil; Sundberg, Carl Johan; Pernow, John; Medson, Koshiar; Kowalski, Jan H.; Rodriguez-Wallberg, Kenny A.; Zheng, Xiaowei; Catrina, Sergiu; Runold, Michael; Ståhlberg, Marcus; Bruchfeld, Judith; Nygren-Bonnier, Malin; Lindholm, Peter	Hyperbaric oxygen for treatment of long COVID-19 syndrome (HOT-LoCO): protocol for a randomised, placebo-controlled, double-blind, phase II clinical trial.	BMJ open	wrong population
2022	Chen, Jiping; Han, Bing; Wu, Chenggang	On the superiority of a combination of aerobic and resistance exercise for fibromyalgia syndrome: A network meta-analysis.	Frontiers in psychology	wrong study design
2022	Souza, Mateus B.; Mascarenhas, Rodrigo O.; Maia, Laisa B.; Fonseca, Letícia S.; Silva, Hytalo J.; de Zoete, Rutger M. J.; McAuley, James H.; Henschke, Nicholas; Oliveira, Vinicius C.	Comparative efficacy and acceptability of non-pharmacological interventions in fibromyalgia: Protocol for a network meta-analysis.	PloS one	wrong study design

2022	Buranruk, Orawan	A randomized clinical trial of self-stretching with and without mindful breathing - immediate effect on pressure pain and range of motion in myofascial pain syndrome.	Journal of bodywork and movement therapies	wrong intervention
2022	Ma, Ji; Zhang, Teng; Li, Xin; Chen, Xiaoyu; Zhao, Qian	Effects of aquatic physical therapy on clinical symptoms, physical function, and quality of life in patients with fibromyalgia: A systematic review and meta-analysis.	Physiotherapy theory and practice	wrong study design
2022	Paoletta, Marco; Moretti, Antimo; Liguori, Sara; Toro, Giuseppe; Gimigliano, Francesca; Iolascon, Giovanni	Efficacy and Effectiveness of Extracorporeal Shockwave Therapy in Patients with Myofascial Pain or Fibromyalgia: A Scoping Review.	Medicina (Kaunas, Lithuania)	wrong study design
2022	Valera-Calero, Juan Antonio; Fernández-de-Las-Peñas, César; Navarro-Santana, Marcos José; Plaza-Manzano, Gustavo	Efficacy of Dry Needling and Acupuncture in Patients with Fibromyalgia: A Systematic Review and Meta-Analysis.	International journal of environmental research and public health	wrong study design
2022	Tehrani, Mohammad Reza; Nazary-Moghadam, Salman; Zeinalzadeh, Afsaneh; Moradi, Ali; Mehrad-Majd, Hassan; Sahebalam, Mohamad	Efficacy of low-level laser therapy on pain, disability, pressure pain threshold, and range of motion in patients with myofascial neck pain syndrome: a systematic review and meta-analysis of randomized controlled trials.	Lasers in medical science	wrong study design
2022	Huang, Yifa; Gao, Mintai; Li, Qiaomin; Zhang, Xuzheng; Chen, Huizhen; Li, Xinglu; Hu, Ping; Zeng, Qingshi	Ultrasound-Guided Dry Needling for Trigger Point Inactivation in the Treatment of Postherpetic Neuralgia Mixed with Myofascial Pain Syndrome: A Prospective and Controlled Clinical Study.	Pain research & management	wrong intervention
2022	Huang, Jiafu; Zhong, Mengting; Wang, Jinghao	Effects of Exercise-Based Interventions on Functional Movement Capability in Untrained Populations: A Systematic Review and Meta-Analysis.	International journal of environmental research and public health	wrong population

2022	Lynæs, Christian; Lynæs, Maria; Simon, Lee S.; Tugwell, Peter; D'Agostino, Maria-Antonietta; Strand, Vibeke; Juhl, Carsten B.; Nielsen, Sabrina M.; De Witt, Maarten; Beaton, Dorcas; Maxwell, Lara J.; Meara, Alexa S.; Christensen, Robin	Physicians' vs patients' global assessments of disease activity in rheumatology and musculoskeletal trials: A meta-research project with focus on reasons for discrepancies.	Seminars in arthritis and rheumatism	wrong study design
2022	Lombardo, Mauro; Feraco, Alessandra; Ottaviani, Morena; Rizzo, Gianluca; Camajani, Elisabetta; Caprio, Massimiliano; Armani, Andrea	The Efficacy of Vitamin D Supplementation in the Treatment of Fibromyalgia Syndrome and Chronic Musculoskeletal Pain.	Nutrients	wrong intervention
2022	Almutairi, Nawaf Masaad; Hilal, Faisal Mohammed; Bashawyah, Ahmed; Dammas, Fatma Al; Yamak Altinpulluk, Ece; Hou, Jin-De; Lin, Jui-An; Varrassi, Giustino; Chang, Ke-Vin; Allam, Abdallah El-Sayed	Efficacy of Acupuncture, Intravenous Lidocaine, and Diet in the Management of Patients with Fibromyalgia: A Systematic Review and Network Meta-Analysis.	Healthcare (Basel, Switzerland)	wrong study design
2022	Allsop, Vivianne L.; Schmid, Arlene A.; Miller, Kristine K.; Slaven, James E.; Daggy, Joanne K.; Froman, Amanda; Kline, Matthew; Sargent, Christy; French, Dustin D.; Ang, Dennis; Van Puymbroeck, Marieke; Schalk, Nancy L.; Bair, Matthew J.	The Pain Outcomes Comparing Yoga vs. Structured Exercise (POYSE) Trial in Veterans With Fibromyalgia: Study Design and Methods.	Frontiers in pain research (Lausanne, Switzerland)	wrong intervention
2022	Migliorini, Filippo; Maffulli, Nicola; Knobe, Matthias; Tenze, Giacomo; Aljalloud, Ali; Colarossi, Giorgia	Pregabalin administration in patients with fibromyalgia: a Bayesian network meta-analysis.	Scientific reports	wrong study design
2022	Pontes-Silva, André	Recommendations for randomised controlled trials on fibromyalgia and myalgic encephalomyelitis.	Autoimmunity reviews	wrong intervention

2022	Soleiman, Farzaneh; Kouhzad Mohamadi, Hosein; Saadat, Maryam; Derisfard, Fateme; Nassadj, Gholamhossein	A protocol for a randomized trial on pain neuroscience education vs. routine physical therapy in people with chronic neck pain.	European journal of translational myology	wrong population
2022	Li, Chong; Sun, Mingyu; Tian, Shiliu	Research Hotspots and Effectiveness of Transcranial Magnetic Stimulation in Pain: A Bibliometric Analysis.	Frontiers in human neuroscience	wrong intervention
2022	Nielsen, Svetlana Solgaard; Skou, Søren T.; Larsen, Anette Enemark; Bricca, Alessio; Søndergaard, Jens; Christensen, Jeanette Reffstrup	The Effect of Occupational Engagement on Lifestyle in Adults Living with Chronic Pain: A Systematic Review and Meta-analysis.	Occupational therapy international	wrong intervention
2022	Scaturro, Dalila; Vitagliani, Fabio; Tomasello, Sofia; Filippetti, Mirko; Picelli, Alessandro; Smania, Nicola; Letizia Mauro, Giulia	Can the Combination of Rehabilitation and Vitamin D Supplementation Improve Fibromyalgia Symptoms at All Ages?	Journal of functional morphology and kinesiology	wrong intervention
2022	Couto, Nuno; Monteiro, Diogo; Cid, Luís; Bento, Teresa	Effect of different types of exercise in adult subjects with fibromyalgia: a systematic review and meta-analysis of randomised clinical trials.	Scientific reports	wrong study design
2022	Morgan, Philip J.; Grounds, Jacqueline A.; Ashton, Lee M.; Collins, Clare E.; Barnes, Alyce T.; Pollock, Emma R.; Kennedy, Stevie-Lee; Rayward, Anna T.; Saunders, Kristen L.; Drew, Ryan J.; Young, Myles D.	Impact of the 'Healthy Youngsters, Healthy Dads' program on physical activity and other health behaviours: a randomised controlled trial involving fathers and their preschool-aged children.	BMC public health	wrong population
2023	Zhu, Ping-An; Xie, Ju-Ying; Liu, Howe; Wen, Youliang; Shao, Yin-Jin; Bao, Xiao	Efficacy of High-Frequency Repetitive Transcranial Magnetic Stimulation at 10 Hz in Fibromyalgia: A Systematic Review and Meta-analysis.	Archives of physical medicine and rehabilitation	wrong study design

2022	Albuquerque, Maria Luiza L.; Monteiro, Diogo; Marinho, Daniel A.; Vilarino, Guilherme T.; Andrade, Alexandro; Neiva, Henrique P.	Effects of different protocols of physical exercise on fibromyalgia syndrome treatment: systematic review and meta-analysis of randomized controlled trials.	Rheumatology international	wrong intervention
2022	Suso-Martí, Luis; Cuenca-Martínez, Ferran; Alba-Quesada, Patricio; Muñoz-Alarcos, Vicente; Herranz-Gómez, Aida; Varangot-Reille, Clovis; Domínguez-Navarro, Fernando; Casaña, José	Effectiveness of Pain Neuroscience Education in Patients with Fibromyalgia: A Systematic Review and Meta-Analysis.	Pain medicine (Malden, Mass.)	wrong study design
2022	Alsouhibani, Ali; Hoeger Bement, Marie	Impaired conditioned pain modulation was restored after a single exercise session in individuals with and without fibromyalgia.	Pain reports	wrong intervention
2022	Murillo-Garcia, Alvaro; Adsuar, Jose C.; Villafaina, Santos; Collado-Mateo, Daniel; Gusi, Narcis	Creative versus repetitive dance therapies to reduce the impact of fibromyalgia and pain: A systematic review and meta-analysis.	Complementary therapies in clinical practice	wrong study design
2022	Dailey, Dana L.; Vance, Carol G. T.; Chimenti, Ruth; Rakel, Barbara A.; Zimmerman, Miriam Bridget; Williams, Jon M.; Sluka, Kathleen A.; Crofford, Leslie J.	The Influence of Opioids on Transcutaneous Electrical Nerve Stimulation Effects in Women With Fibromyalgia.	The journal of pain	wrong intervention
2022	Wu, Jinlong; Chen, Ziyan; Zheng, Kangyong; Huang, Weiwei; Liu, Fang; Lin, Jian; Ren, Zhanbing	Benefits of Exergame Training for Female Patients With Fibromyalgia: A Systematic Review and Meta-Analysis of Randomized Controlled Trials.	Archives of physical medicine and rehabilitation	wrong study design
2022	Graham, Michael; Azevedo, Liane; Wright, Matthew; Innerd, Alison L.	The Effectiveness of Fundamental Movement Skill Interventions on Moderate to Vigorous Physical Activity Levels in 5- to 11-Year-Old Children: A Systematic Review and Meta-Analysis.	Sports medicine (Auckland, N.Z.)	wrong population

2022	Kundakci, Burak; Kaur, Jaspreet; Goh, Siew Li; Hall, Michelle; Doherty, Michael; Zhang, Weiya; Abhishek, Abhishek	Efficacy of nonpharmacological interventions for individual features of fibromyalgia: a systematic review and meta-analysis of randomised controlled trials.	Pain	wrong study design
2022	Climent-Sanz, Carolina; Valenzuela-Pascual, Fran; Martínez-Navarro, Oriol; Blanco-Blanco, Joan; Rubí-Carnacea, Francesc; García-Martínez, Ester; Soler-González, Jorge; Barallat-Gimeno, Eva; Gea-Sánchez, Montserrat	Cognitive behavioral therapy for insomnia (CBT-i) in patients with fibromyalgia: a systematic review and meta-analysis.	Disability and rehabilitation	wrong study design
2022	Jamison, Robert N.; Curran, Samantha; Wan, Limeng; Ross, Edgar L.; Gilligan, Christopher J.; Edwards, Robert R.	Higher Pain Sensitivity Predicts Efficacy of a Wearable Transcutaneous Electrical Nerve Stimulation Device for Persons With Fibromyalgia: A Randomized Double-Blind Sham-Controlled Trial.	Neuromodulation : journal of the International Neuromodulation Society	wrong intervention
2022	Graham, Andrew; Ryan, Cormac G.; MacSween, Alasdair; Alexanders, Jenny; Livadas, Nick; Oatway, Sarah; Atkinson, Greg; Martin, Denis J.	Sensory discrimination training for adults with chronic musculoskeletal pain: a systematic review.	Physiotherapy theory and practice	wrong study design
2017	Celebi, E; Ataoglu, S; Ataoglu, BB; Ankarali, H; Pasin, O; Olmez, SB	The Investigation of Effects of Pregabalin and Duloxetine Treatment According to Personality Characteristics with Fibromyalgia Patients	Psychiatry and clinical psychopharmacology	wrong intervention
2022	Li, S; Zhang, Z; Jiao, Y; Jin, G; Wu, Y; Xu, F; Zhao, Y; Jia, H; Qin, Z; Zhang, Z; et al.	An assessor-blinded, randomized comparative trial of transcutaneous auricular vagus nerve stimulation (taVNS) combined with cranial electroacupuncture vs. citalopram for depression with chronic pain	Frontiers in psychiatry	wrong intervention

2016	NCT02695875	Comparative Study About the Effect of Aquatic Therapy vs Land-based Therapy in Women With Fibromyalgia	https://clinicaltrials.gov/show/NCT02695875	uncompleted study
2008	NCT00615654	Aquatic Physical Therapy in Fibromyalgia	https://clinicaltrials.gov/show/NCT00615654	uncompleted study
2015	NCT02458326	Aerobic Training Effect on the Improvement of Pain Perception in Patients With Fibromyalgia and Migraine	https://clinicaltrials.gov/show/NCT02458326	uncompleted study
2020	NCT04673058	Effectiveness of Spinal Manipulation in Fibromyalgia	https://clinicaltrials.gov/show/NCT04673058	uncompleted study
2018	NCT03630757	Effects of Manual Therapy in Fibromyalgia Syndrome	https://clinicaltrials.gov/show/NCT03630757	uncompleted study
2016	NCT02864524	Manipulative and Massage Therapy in the Lower Thoracic and Cervical Spine in Subjects With Fibromyalgia Syndrome	https://clinicaltrials.gov/show/NCT02864524	uncompleted study
2021	NCT04835077	Aerobic Exercises and Postural Stabilization Exercises in Fibromyalgia Syndrome	https://clinicaltrials.gov/show/NCT04835077	uncompleted study
2004	NCT00086047	Coping Skills Training for Adolescents With Fibromyalgia	https://clinicaltrials.gov/show/NCT00086047	uncompleted study
2013	NCT02004405	Heart Rate Variability in Fibromyalgia - Effects of Strengthening Exercises	https://clinicaltrials.gov/show/NCT02004405	uncompleted study
2020	NCT04571528	Effectiveness of VIRTUAL FIBROWALK STUDY	https://clinicaltrials.gov/show/NCT04571528	uncompleted study

2005	ISRCTN21694164	A randomised controlled trial to evaluate the effectiveness of a combined education and pool-based exercise programme and usual care in the treatment of patients with fibromyalgia syndrome	http://www.who.int/trialsearch/Trial2.aspx?TrialID=ISRCTN21694164	uncompleted study
2012	NCT01547195	Effects of Swimming in the Treatment of Fibromyalgia	https://clinicaltrials.gov/show/NCT01547195	uncompleted study
2020	NCT04554784	Effectiveness of Bowen Therapy for Pain Management in Patients With Fibromyalgia	https://clinicaltrials.gov/show/NCT04554784	uncompleted study
2019	NCT03997695	Effects of Core Stabilization Exercise Plus Kinesio Taping in Woman With Fibromyalgia	https://clinicaltrials.gov/show/NCT03997695	uncompleted study
2011	NCT01278641	Men With Fibromyalgia or Chronic Widespread Pain - Effect of Exercise on Symptoms and Body Functions	https://clinicaltrials.gov/show/NCT01278641	uncompleted study
2021	KCT0006316	Comparison of low back pain management between artificial intelligence-based low back pain management application and conventional low back pain management in adults with low back pain	http://www.who.int/trialsearch/Trial2.aspx?TrialID=KCT0006316	uncompleted study
2023	NCT05710939	Effect of Scapular Stabilization Exercise Training on Posture and Pain in Fibromyalgia Patients	https://clinicaltrials.gov/show/NCT05710939	uncompleted study
2022	ACTRN12622000573752	Functional assessment of orthopedic patients undergoing selected methods of physiotherapy	https://trialsearch.who.int/Trial2.aspx?TrialID=ACTRN12622000573752	uncompleted study

2022	IRCT20210811052138N4	Comparison of theraband and plyometrics training on balance and motor performance in footballers	https://trialsearch.who.int/Trial2.aspx?TrialID=IRCT20210811052138N4	uncompleted study
2016	ACTRN12616000390482	Impact of balance training on specific athletic performance in adolescents practicing football	http://www.who.int/trialsearch/Trial2.aspx?TrialID=ACTRN12616000390482	uncompleted study
2017	ACTRN12617000835347	Changes in movement quality and physical performance in response to an 8 week individualised movement quality assessment informed exercise intervention in apparently healthy adults: a randomised trial	http://www.who.int/trialsearch/Trial2.aspx?TrialID=ACTRN12617000835347	wrong intervention
2023	NCT05719493	Effectiveness and Health Benefits of a Nutritional, Chronobiological and Physical Exercise Intervention in Fibromyalgia and Chronic Fatigue Syndrome (SYNCHRONIZE +)	https://clinicaltrials.gov/show/NCT05719493	wrong intervention
2015	ACTRN12615001316594	Impact of NEURAC stability training on specific athletic performance and abdominal muscle activity among children and adolescents practicing volleyball	http://www.who.int/trialsearch/Trial2.aspx?TrialID=ACTRN12615001316594	wrong population
